# Association of MicroRNA-196a2 Variant with Response to Short-Acting β2-Agonist in COPD: An Egyptian Pilot Study

**DOI:** 10.1371/journal.pone.0152834

**Published:** 2016-04-04

**Authors:** Manal S. Fawzy, Mohammad H. Hussein, Eman Z. Abdelaziz, Hussain A. Yamany, Hussein M. Ismail, Eman A. Toraih

**Affiliations:** 1 Department of Medical Biochemistry, Faculty of Medicine, Suez Canal University, Ismailia, Egypt; 2 Department of Chest Diseases, Faculty of Medicine, Cairo University, Giza, Egypt; 3 Department of Pharmacology, Faculty of Medicine, Suez Canal University, Ismailia, Egypt; 4 Department of Medicine, College of Medicine, Taibah University, Almadinah Almunawwarah, Kingdom of Saudi Arabia; 5 Department of Cardiology, Faculty of Medicine, Suez Canal University, Ismailia, Egypt; 6 Department of Histology and Cell Biology (Genetics Unit), Faculty of Medicine, Suez Canal University, Ismailia, Egypt; The University of Hong Kong, CHINA

## Abstract

Chronic obstructive pulmonary disease (COPD) is a multifactorial chronic respiratory disease, characterized by an obstructive pattern. Understanding the genetic predisposition of COPD is essential to develop personalized treatment regimens. MicroRNAs (miRNAs) are small, endogenous, non-coding RNAs that modulate the expression levels of specific proteins based on sequence complementarity with their target mRNA molecules. Emerging evidences demonstrated the potential use of miRNAs as a disease biomarker. This pilot study aimed to investigate the association of the MIR-196a2 rs11614913 (C/T) polymorphism with COPD susceptibility, the clinical outcome and bronchodilator response to short-acting β_2_-agonist. Genotyping of rs11614913 polymorphism was determined in 108 COPD male patients and 116 unrelated controls using real-time polymerase chain reaction technology. *In silico* target prediction and network core analysis were performed. COPD patients did not show significant differences in the genotype distribution (*p* = 0.415) and allele frequencies (*p* = 0.306) of the studied miRNA when compared with controls. There were also no associations with GOLD stage, dyspnea grade, disease exacerbations, COPD assessment test for estimating impact on health status score, or the frequency of intensive care unit admission. However, COPD patients with CC genotype corresponded to the smallest bronchodilator response after Salbutamol inhalation, the heterozygotes (CT) had an intermediate response, while those with the TT genotype showed the highest response (*p* < 0.001). In conclusion MIR-196a2 rs11614913 polymorphism is associated with the bronchodilator response of COPD in our sample of the Egyptian population, generating hypothesis of the potential use of MIR-196a2 variant as a pharmacogenetic marker for COPD.

## Introduction

Chronic obstructive pulmonary disease (COPD) is a main cause of morbidity and mortality worldwide and represents a large and increasing burden to the health care system [1, 2]. COPD prevalence is continually increasing and it is predicted to become the third leading cause of death worldwide by 2020 [3]. It is a heterogeneous disease defined by the persistent airflow limitation that is usually progressive and an enhanced chronic inflammatory response of the lung to noxious particles and gases [4]. COPD is characterized by two distinct and frequently coexisting aspects: small airway abnormalities and parenchymal destruction [or emphysema] [5]. It often coexists with other comorbidities that may have a significant prognostic impact [4, 6]. Genomic association studies are currently emerging to discover genes and molecular pathways involved in COPD pathogenesis [7]. Understanding the genetic predisposition of COPD is essential to develop early personalized treatment regimens [4].

MicroRNAs (miRNAs) are small non-coding RNA molecules that modulate the levels of protein-coding genes [8]. Bioinformatics data indicated that a single miRNA could bind to hundreds of messenger RNA targets causing their degradation or inhibition of translation [9]. Thus, they are virtually involved in every cellular processes, including proliferation, differentiation, development and apoptosis [10]. MiRNAs have been identified in different human tissues and body fluids, as serum, plasma, sputum, saliva, bronchial lavage, and pleural fluid [11]. Altered miRNA expression profile have been implicated in multiple pulmonary diseases, including COPD [3, 12–14], pulmonary tuberculosis [15], smoking [16, 17], asthma [18,19], idiopathic pulmonary fibrosis [20–22], cystic fibrosis [23], bronchopulmonary dysplasia [24], acute respiratory distress syndrome [25], ventilator-induced lung injury [26] and lung cancer [27–29].

Aberrant expression can be caused by single nucleotide polymorphisms (SNPs) in miRNA genes [8, 27]. These SNPs may alter miRNA processing, expression, and/or miRNA–mRNA interactions, resulting in different functional events which may play a crucial role in the development and progression of pulmonary diseases [30]. In human, a SNP (rs11614913; C/T) was identified in MIR-196a2 (*hsa-miR-196a2*) gene [31]. Reports have demonstrated that this particular polymorphism could alter mature miR-196a2 expression and binding to target mRNAs [32, 33]. Thus, this preliminary study was conducted to investigate the association of rs11614913 polymorphism with susceptibility to COPD disease, and to further assess their impact on the clinical outcome and bronchodilator response (BDR) to short-acting β_2_-agonist.

## Materials and Methods

### Study participants

The study population included 116 COPD male patients, whose eight patients declined to give blood samples and 116 healthy matched controls. Patients were recruited from Suez Canal University Hospital, and Port-Said Chest Hospital, Egypt, during the period between May 2013 and August 2014. Detailed history was obtained using a structured interview, including occupation, smoking, and family history of COPD. Patients were diagnosed and assessed according to the Global Initiative for Chronic Obstructive Lung Disease (GOLD) criteria [4]. Exacerbation episodes and hospital admission during the last year, along with the history of regular medications used for COPD were obtained. Severity of dyspnea was assessed using the Modified Medical Research Council (mMRC) scale [34], while patients' symptoms and the impact of COPD disease on health status were assessed using the COPD Assessment Test (CAT) score [35, 36]. Chest radiographs were performed to exclude other causes of airflow limitation. Controls were individuals who admitted to the hospital for an elective surgery excluding thoracic and abdominal one and subjected to our assessment (blood sample tests, spirometry, and chest X-ray) as part of routine preoperative workup. This study was conducted in accordance with the guidelines in the Declaration of Helsinki and approved by the Medical Research Ethics Committee of Faculty of Medicine, Suez Canal University (approval no. 2511). An informed written consent was obtained from all participants.

### Pulmonary function tests

Spirometry was performed for all participants using an electronic spirometer (BTL-08 Spiro Pro system; BTL) in accordance with the guidelines of the American Thoracic Society/European Respiratory Society (ATS/ERS) consensus to evaluate their baseline pulmonary function [37]. In patients, bronchodilator forced spirometry was repeated 15 minutes after administration of 400 μg of inhaled Salbutamol (Ventolin; GlaxoSmithKline) [38].

### SNP identification

Genomic DNA was extracted from venous blood using QIAamp DNA Blood Mini kit (Cat. No. 51104, QIAGEN, Germany) according to the manufacturer's instructions. Extracted DNA concentration and purity were measured by NanoDrop ND-1000 (NanoDrop Tech., Inc. Wilmington, DE, USA). DNA samples of patients and controls were genotyped for the hsa-miR-196a2 polymorphism (*rs11614913*) using Real-Time polymerase chain reaction technology in accordance with the Minimum Information for Publication of Quantitative Real-Time PCR Experiments (MIQE) guidelines [39, 40]. PCR was performed in a 25-μl reaction volume containing genomic DNA (20 ng) diluted to 11.25μL with DNase-RNase-free water, 12.5 μl Taqman^®^ Universal PCR Master Mix; No AmpErase UNG (2x) and 1.25 μl 20x TaqMan^®^ SNP Genotyping Assay Mix (Cat. No. 4351379, Applied Biosystems, Foster City, USA). Genotyping was performed blinded to case/control status. Appropriate negative controls were used. Real-time PCR amplification was performed on StepOne^™^ Real-Time PCR System (Applied Biosystems, Foster City, USA) using the following conditions: an initial hold (95°C for 10 min) followed by a 40-cycle two-step PCR (95°C denaturation for 15 s and annealing/extension 60°C for 1 min). Allelic discrimination was called by the SDS software version 1.3.1 (Applied Biosystems). Ten per cent of the samples were randomly selected for the repeated assays and the results were 100% concordance.

### *In silico* gene target prediction for miR-196a2 and network core analysis

**Fig 1:**
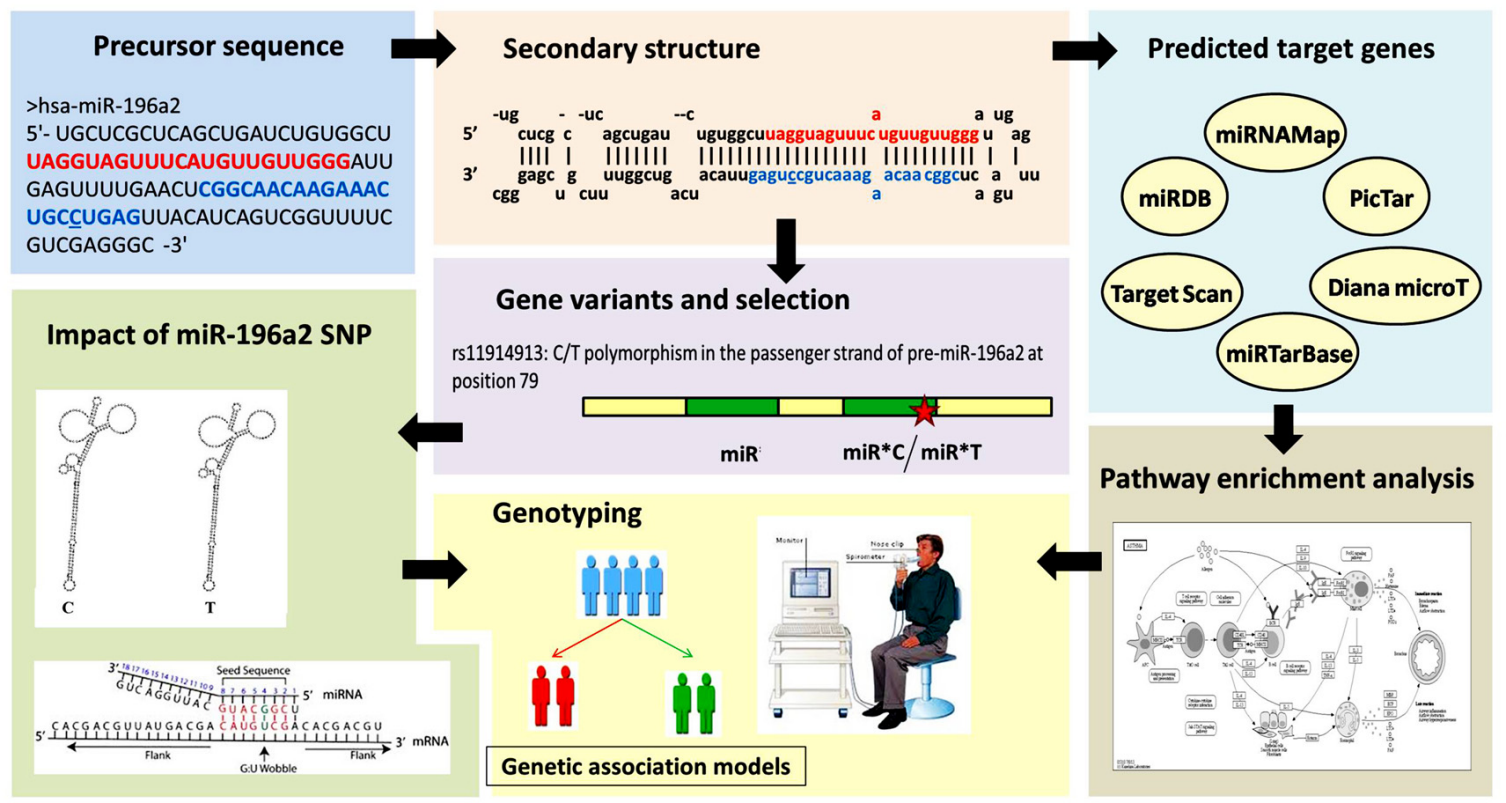
Workflow of *in silico* data analysis. Gene and microRNA sequence and structure were retrieved from miRBase database (http://microrna.sanger.ac.uk/targets/v3/). Multiple computational prediction tools were employed to identify miR-196a2 target genes (in 3'URT, 5'UTR, and CDS) as miRDB, miRNAMap, TargetScanHuman v6.2, miRTarBase v18 and DIANA-microT-CDS v5.0 databases. Result intersection, statistical validation, and filtration of the putative miRNA targets were applied to reduce the false positive prediction rate. The predicted miRNA target genes were analyzed for gene ontology (GO) terms and KEGG enrichment pathway analysis using DIANA-miRPath v2.0 web-server (http://diana.imis.athenainnovation.gr/DianaTools/index.php?r=mirpath/index) and miRTar Human tool (http://miRTar.mbc.nctu.edu.tw/). MiRNA-196a2-disease association was explored using a miRPub server (http://www.microrna.gr/mirpub/). Gene variations and frequencies in various populations were obtained from Ensembl (http://www.ensembl.org/) and miRdSNP databases (http://mirdsnp.ccr.buffalo.edu/). The Impact of the SNP on secondary structure was predicted based on the minimum free energy using miRNAMap 2.0 and RNAfold server. Comparative functional analysis between predicted target gene sets in wild and mutant variants were performed using miRmut2Go. SNP identification in the study groups aimed to assess its association with disease risk, severity and pulmonary function.

The workflow for the conducted *in silico* analysis is illustrated in Fig 1. Analysis of predicted miRNA target genes was performed using multiple online software packages including miRTarBase version 18 (http://mirtarbase.mbc.nctu.edu.tw/), miRDB (http://mirdb.org/miRDB/), miRBase (http://microrna.sanger.ac.uk/targets/v3/), TargetScanHuman v6.2 (http://www.targetscan.org/), DIANA-microT-CDS v5.0 (http://diana.cslab.ece.ntua.gr/pathways/), PicTar (http://pictar.mdc-berlin.de/), other microRNA databases (http://www.microrna.org/microrna/home.do) (S1 Table). In order to identify Kyoto Encyclopedia of Genes and Genomes (KEGG) pathways associated with our set of genes, we used DAVID (Database for Annotation, Visualization and Integrated Discovery) software and obtained the list of functional enriched pathways (S2 Table) [41]. To identify genes and networks involved in the pathogenesis of COPD, we used the data from the Ingenuity Pathway Analysis platform (Ingenuity Systems Inc, Redwood City, CA) [42] and literature review (S3 Table) [14, 43–44]. In order to check if SNP rs11614913 in *miR196a2* affected its secondary structure, we studied the minimum free energy for optimal secondary structure of this variant for miRNA using RNAfold server (http://rna.tbi.univie.ac.at/cgi-bin/RNAfold.cgi).

### Statistical analysis

Statistical analysis was carried out using the “Statistical Package for Social Sciences (SPSS) for windows” software, version 20. The appropriate sample size and power calculations were carried out using Quanto software package version 1.2.4 (University of Southern California; http://biostats.usc.edu/software). Categorical variables were compared using chi-square (χ^2^) test, while the Studenť s t test and one way analysis of variance (ANOVA) were used to compare continuous variables between two groups, and for more than two groups, respectively, in case the data distribution was concordant with normal distribution (Shapiro-Wilk test) and after checking variance homogeneity (Levene test), followed by appropriate multiple comparison test (post-hoc Newman-Keuls test). If the data did not meet the criteria mentioned above, the non-parametric Mann-Whitney (MW) test for comparing two groups and Kruskal-Wallis (KW) test for comparing more than two groups followed by Dunn's test, were applied. The allelic frequencies were calculated by direct counting the alleles. Genotype distributions in patients and controls were evaluated for departure from Hardy–Weinberg equilibrium (HWE) by the Online Encyclopedia for Genetic Epidemiology (OEGE) software (http://www.oege.org/software/hwe-mr-calc.shtml). The goodness of fit for the Chi square test was applied to compare the expected versus observed distribution of genotypes. Odds ratios (OR) with a 95% confidence interval (CI) were calculated for the following genetic models; allelic model (T versus C), homozygote comparison (TT versus CC), heterozygote comparison (CT versus CC), dominant model (TT+CT versus CC), and recessive model (TT versus CT+CC). Binary logistic regression analysis was performed to adjust potential confounders (obesity, smoking, family history of COPD) with the polymorphism as an independent variable. A two-tailed *P-*value of 0.05 was considered statistically significant. The Brinkman smoking index was calculated as the number of cigarettes smoked per day × number of years. Response to salbutamol was expressed in three ways: [1] absolute change in forced expiratory volume in first second FEV_1_ [BDRABS = postbronchodilator FEV_1_ − prebronchodilator FEV_1_], [2] change in FEV_1_ as a percent of baseline FEV_1_ [BDRBASE = (postbronchodilator FEV_1_ − prebronchodilator FEV_1_) / (prebronchodilator FEV_1_)× 100], and [3] change in FEV_1_ as a percent of predicted FEV_1_ [BDRPRED = (postbronchodilator FEV_1_ − prebronchodilator FEV_1_) / (predicted FEV_1_)× 100] [45, 46].

## Results

### *In silico* data analysis

**Fig 2:**
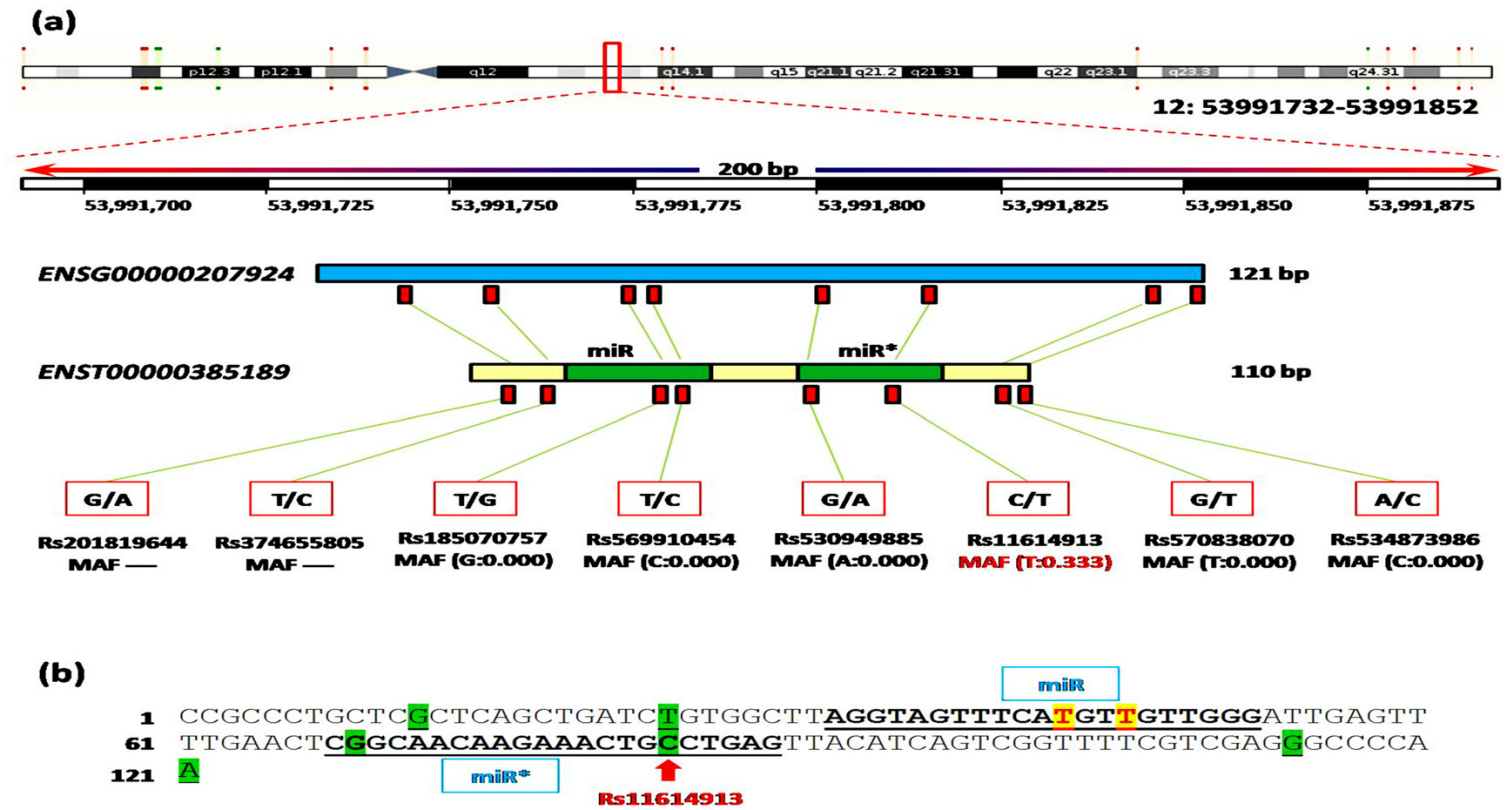
Mapping MIR196A2 gene variations. (a) Human hsa-miR-196a2 gene has 2 mature miRNA variants located within the sequence of the mature miRNA and 6 non-coding transcript variants. Chromosomal location and coordinates in base pair are derived from human genome assembly GRCh38. Eight gene variations were retrieved from Ensembl. Variant ID, alternative nucleotides, and minor allele frequency (MAF) are shown. All polymorphisms were very rare (MAF<0.1), except the studied variant. (b) Mature miR and miR* sequences are underlined. Green highlighted nucleotides are non-coding transcript variants in pre-miR-196a2. Yellow highlighted nucleotides are mature miRNA variants at 5p arm. Red arrow indicates the studied common variant.

Human hsa-miR-196a2 gene (*MIR196A2; ENSG00000207924*) is located within the region of homeobox (*HOX*) gene clusters on chromosome 12q13.13 (*human genome assembly GRCh38*: position 53991732–53991852 forward strand, 121 bp long). The gene produces a single premature transcript (*ENST00000385189*) of 110 bases long. Within MIR196A2 gene, eight SNPs had been identified. Our common variant exists in the passenger strand of miR-196a2; Fig 2. We found that this SNP in pre-miR196a2 had no dramatic effect on its secondary structure computed based on the minimum free energy using miRNAMap 2.0 (http://mirnamap.mbc.nctu.edu.tw/) and RNAfold server. In addition, comparative functional analysis between predicted target gene sets in wild and mutant variants using miRmut2Go browser (http://compbio.uthsc.edu/miR2GO), showed complete similarity as the SNP is located outside the seed region essential for binding to their targets.

**Fig 3:**
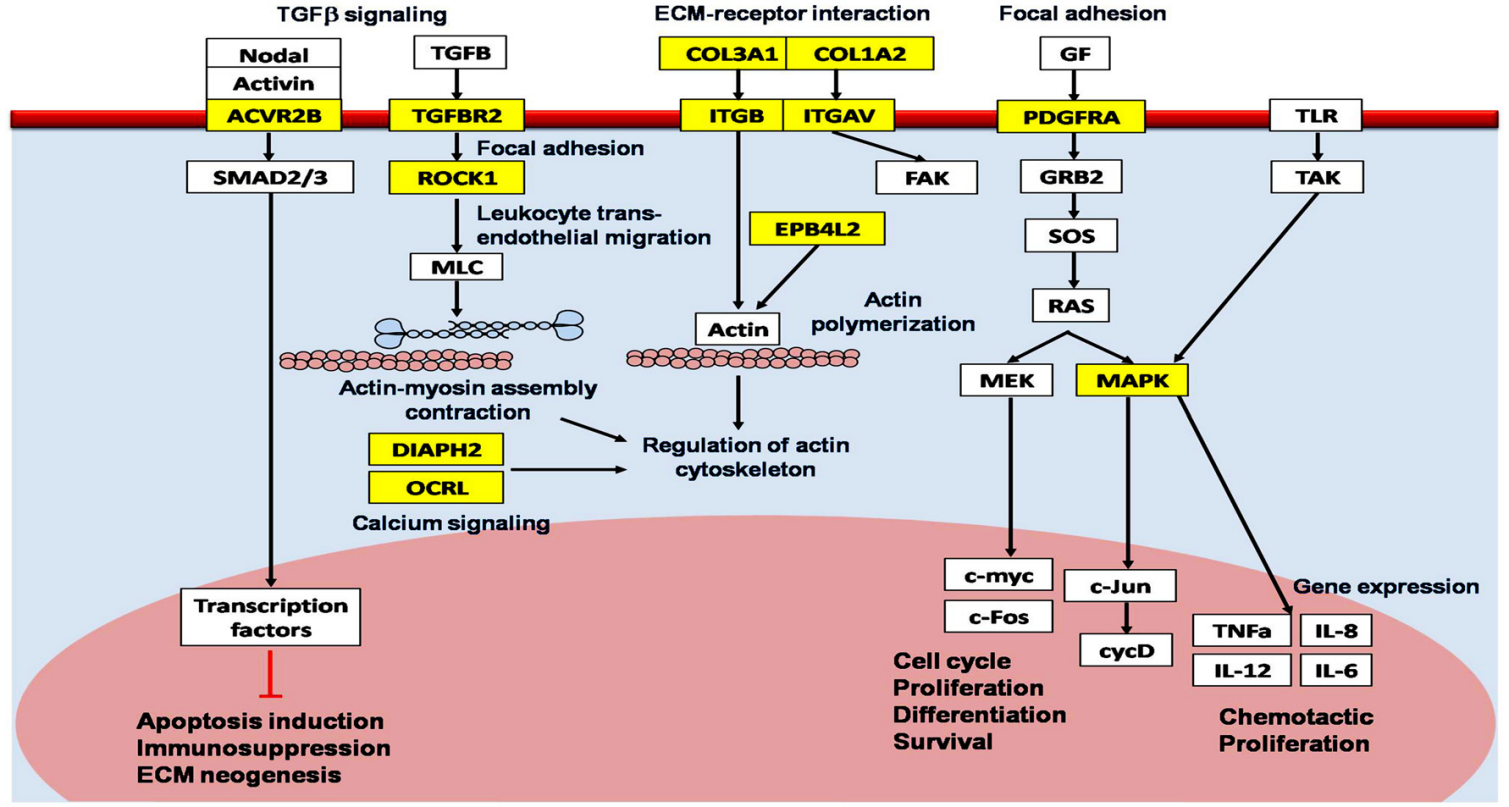
MicroRNA-196a2 predicted target gene products with putative roles in chronic obstructive pulmonary disease. The diagram was manually curated by combining *in silico* analysis of predicted hsa-miR-196a2-targeted signaling pathways involved in the pathogenesis of COPD and review of previous literatures. KEGG pathways (blue); predicted target genes of miR-196a2 (yellow box); other genes in the pathway network (white box); activate (black arrow); inhibit (red line). *TGFB* transforming growth factor beta, *ACVR2B* activin A receptor type IIB, SMAD2/3, human mothers against decapentaplegic homolog 2/3, *ECM* extracellular matrix, *TGFBR2* TGFB receptor 2, *ROCK1* rho-associated coiled-coil containing protein kinase 1, *MLC* myosin light chain, *DIAPH2* diaphanous homolog 2, *OCRL* oculocerebrorenal syndrome of Lowe, *COL3A1* collagen type 3 alpha 1, *ITGB* integrin beta, *ITGAV* integrin alpha V, *FAK* focal adhesion kinase, *EPB4L2* erythrocyte membrane protein band 4-like 2, *GF* growth factor; *PDGFRA* platelet-derived growth factor receptor alpha polypeptide, *GRB2* growth factor receptor binding protein 2, *mSOS* mammalian son of sevenless, *RAS* rat associated sarcoma, *MEK* mitogen-activated protein kinase and extracellular regulated kinase kinase, *c-myc* cellular oncogene originally identified as the transforming determinant of avian myelocytomatosis virus, *c-Fos* cellular oncogene homologue to that of FINKEL induce murine osteosarcoma, *MAPK* mitogen-activated protein kinase, *c-Jun* cellular Proto-oncogene protein Jun, *cycD* cyclin D, *TLR* Toll like receptor, *TAK* TGF-beta activated kinase, *TNFa*, Tumor necrosis factor alpha, *IL-6* interleukin-6.

*In silico* analysis using multiple microRNA databases predicted more than 800 target genes for miR-196a2. Functional annotation clustering of these gene sets revealed that they are involved in multiple functional pathways involved in COPD pathogenesis, including TGFβ (transforming growth factor beta) signaling, immune cell trafficking, calcium signaling, phosphotadidylinositol signaling, actin cytoskeleton regulation, focal adhesion, cell-to-cell signaling, tight junction, mTOR (mammalian target of rapamycin) signaling, apoptosis, tissue remodeling, and wound repair pathways. The pathway diagram in Fig 3 describes the potential relationship between some of the previously-validated and predicted miR-196a2 genes incorporated into KEGG pathways related to COPD pathogenesis (S4 Table).

### Characteristics of the study population

Baseline characteristics and pulmonary function parameters of COPD patients and controls are summarized in Table 1. Patients showed more frequent smoking history and lower baseline pulmonary function as expected.

**Table 1:** Clinical and functional characteristics of COPD patients and controls.

Variables	COPD(*n* = 116)	Controls(*n* = 116)	*P* value
**Mean age, years**	58.8 ± 6.2	57.4 ± 5.9	0.596
**Age categories, (%)**			
< 60	32 (27.6)	40 (34.5)	0.257
≥ 60	84 (72.4)	76 (65.5)	
**Mean BMI, Kg/m**^**2**^	24.6 ± 7.2	23.5 ± 6.8	0.541
**Smoking status, (%)**			
Smoker ^a^	39 (33.6)	20 (17.2)	**<0.001**
Ex-smoker ^a^	64 (55.2)	46 (39.7)	
Non-smoker	13 (11.2)	50 (43.1)	
**Brinkman smoking index**	1341 ± 892	748 ± 301	**<0.001**
**Positive Family history of COPD**	73 (63.0)	72 (62.1)	0.892
**GOLD stage**			
Grade 2	34 (29.3)		
Grade 3	50 (43.1)		
Grade 4	32 (27.6)		
**Dyspnea severity**			
Grade 2	19 (16.4)		
Grade 3	67 (57.8)		
Grade 4	30 (25.8)		
**CAT score, mean**	21.3 ± 8.21		
**Exacerbations ≥ 2 times last year, (%)**	56 (48.3)		
**ICU admission last year, (%)**	32 (27.6)		
**Regular medications**			
SABA	13 (11.2)		
ICS + SABA	26 (22.4)		
ICS + LABA	26 (22.4)		
ICS + SABA + LABA	41 (35.3)		
**Pre-bronchodilator parameters**			
FEV_1_ (% predicted)	40.4 ± 14.6	91.9 ± 11.5	**0.011**
FVC (% predicted)	55.2 ± 19.6	87.1 ± 12.6	**<0.001**
FEV_1_/FVC	57.8 ± 7.71	86.0 ± 5.90	**0.004**
**Post-bronchodilator parameters**			
FEV_1_ (% predicted)	42.2 ± 16.4		
FEV_1_/FVC	58.8 ± 7.1		
**BDR**			
BDRABS (mL)	49.6 ± 44		
BDRPRED (%)	1.8 ± 1.5		
BDRBASE (%)	4.3 ± 3.6		

Values are shown as mean ± standard deviation or as *n* (%). *COPD*, *chronic* obstructive pulmonary disease, *BMI*, *body* mass index, *Brinkman smoking index* number of cigarettes per day times the number of years, *CAT*, COPD Assessment Test for estimating impact on health status, *ICU* intensive care unit, *SABA* short-acting β_2_-agonist, *LABA* Long- acting β_2_-agonist, *ICS* inhaled corticosteroids. Spirometry results before bronchodilatation (*pre-BD*) and after bronchodilatation (*post-BD*). *FEV*_*1*_ Forced expiratory volume in the first second, *FVC* forced vital capacity, *BDR* bronchodilator response, *BDRABS* absolute change in FEV_1_ = (post-BD FEV_1_ –pre-BD FEV_1_), *BDRBASE* change in FEV_1_ as a percent of baseline FEV_1_ = [(post-BD FEV_1_ –pre-BD FEV_1_) / (pre-BD FEV_1_)× 100], *BDRPRED* change in FEV_1_ as a percent of predicted FEV_1_ = [(post-BD FEV_1_ –pre-BD FEV_1_) / (predicted FEV_1_) × 100]. Chi-square test followed by Tukey Style Multiple Comparisons of Proportions and Mann-Whitney U test, were used. Bold values indicate statistically significant at *p* < 0.05,

^**a**^ Indicates significant difference from non-smoker group at *p* < 0.05.

### MIR-196a2 genotype analysis in the study population

Genotyping of the MIR-196a2 rs11614913 (C/T) polymorphism revealed no significant difference between COPD patients and controls under all genetic association models (Table 2). The distribution of genotypes in both study groups was found in accordance with those expected by the Hardy Weinberg equilibrium (*p* > 0.05). The frequency of C allele was more common in our Egyptian population representing (0.75 and 0.71 in patients and controls, respectively).

**Table 2:** Genotype and allele frequencies of *hsa-miR-196a2* (rs11614913) polymorphism in COPD patients and controls.

Genetic model	Genotype	Patients (n = 108)	Controls (n = 116)	*P* value	Crude OR (95% CI)	Adjusted ^a^ OR (95% CI)
**HWE *P* value**		0.700	0.362			
**Co-dominant model** ^b^	**CC**	60 (55.5)	60 (51.8)	0.415	1.0	1.0
	**CT**	42 (38.9)	44 (37.9)		0.9 (0.55–1.66)	0.5 (0.17–1.7)
	**TT**	6 (5.6)	12 (10.3)		0.5 (0.18–142)	1.0 (0.16–3.2)
**Dominant model**	**CC**	60 (55.5)	60 (51.8)	0.565	1.0	1.0
	**CT + TT**	48 (44.5)	56 (48.2)		0.8 (0.4–1.8)	0.7 (0.5–1.3)
**Recessive model**	**CC + CT**	102 (94.4)	104 (89.7)	0.187	1.0	1.0
	**TT**	6 (5.6)	12 (10.3)		0.5 (0.18–1.41)	0.6 (0.12–1.24)
**Allelic model**	**C**	162 (75)	164 (70.7)	0.306	1.0	1.0
	**T**	54 (25)	68 (29.3)		0.8 (0.53–1.22)	0.5 (0.17–1.8)

Values are shown as number (%). HWE *P*; *p* value of Hardy-Weinberg equilibrium. Chi square (χ^2^) test was used. OR (95% CI), odds ratio and confidence interval.

^**a**^ adjusted for confounding factors (obesity, smoking, family history of COPD). Adjusted OR for alleles was calculated as presence versus absence of this particular allele.

^**b**^ represented both heterozygote and homozygote comparison models.

### MIR-196a2 gene polymorphism in relation to clinical characteristics of COPD patients

We followed the Global Initiative for Chronic Obstructive Lung Disease criteria for evaluation of our COPD patients. Assessment of patients included many symptoms and signs as dyspnea, wheezes, cough, sputum production, exacerbations, hospital admission, comorbidities, complications, degree of airflow limitations, etc. For comprehensive assessment of these symptoms, data were collectively used to determine the severity of the disease, its impact on patient's health status, and the risk of future events in the form of the modified British Medical Research Council scale [34], COPD Assessment Test score [35, 36], and GOLD stage [4]. We did not observe any significant association of MIR-196a2 genotypes with these previously mentioned parameters or the frequency of intensive care unit admission (Table 3).

**Table 3:** Disease characteristics and bronchodilator response in COPD patients (n = 108) according to *hsa-miR-196a2* polymorphism.

Characteristics	MIR-196a2 genotypes			*P value*	OR (95%CI) CT/TT *ve*. CC
	CC	CT	TT		
**Total number** (%)	60 (55.5)	42 (38.9)	6 (5.6)		
**Mean age**, years	57.4± 6.7	59.8 ± 4.3	58.3 ± 9.7	0.151	
**Mean BMI**, Kg/m^2^	23.7 ± 5.2	25.5 ± 6.2	23.4 ± 5.8	0.262	
**Brinkman smoking index**	1134 ± 698	1426 ± 765	1245 ± 178	0.128	
**Disease severity**					
GOLD stage ≥3	48 (80)	22 (52.4)	6 (100)	0.354	0.73 (0.40–1.33)
CAT score >20	34 (56.7)	22 (52.4)	0 (0)	0.195	0.81 (0.42–1.56)
MMRC scale ≥3	50 (83.3)	34 (80.9)	6 (100)	0.944	1.00 (0.57–1.76)
Exacerbations ≥2	30 (50)	22 (52.3)	0 (0)	0.222	0.92 (0.47–1.89)
ICU admission	12 (20)	18 (42.8)	0 (0)	0.077	1.88 (0.82–4.27)
**Pulmonary function parameters**					
Pre- FEV1 (% predicted)	36.5 ± 13.8	45.8 ± 17.7^a^	40.3 ± 6.2	**0.012**	
Post- FEV1 (% predicted)	37.5 ± 14.01	48.5 ± 18.7 ^a^	43.6 ± 6.7	**0.003**	
BDRABS (mL)	28.6 ± 39.1	73.3 ± 33.2 ^a^	96.6 ± 37.8 ^a^	**<0.001**	
BDRPRED (%)	1.01 ± 1.4	2.66 ± 1.2 ^a^	3.3 ± 0.9 ^a^	**<0.001**	
BDRBASE (%)	2.91 ± 3.7	5.69 ± 2.5 ^a^	8.2 ± 2.2 ^a^	**<0.001**	

Values are shown as mean ± standard deviation or as *n* (% within genotype). *BMI* body mass index, *Brinkman smoking index* number of cigarettes per day times the number of years, *CAT* COPD Assessment Test for estimating impact on health status, *ICU* intensive care unit, *Pre-FEV*_*1*_ pre-bronchodilator forced expiratory volume in the first second, *Post-FEV*_*1*_ post-bronchodilator, *BDRABS* absolute change in FEV_1_ (post-BD FEV_1_ –pre-BD FEV_1_), *BDRPRED* change in FEV_1_ as a percent of predicted FEV_1_ [= [(post-BD FEV_1_ –pre-BD FEV_1_) / predicted FEV_1_] × 100], *BDRBASE* change in FEV_1_ as a percent of baseline FEV1 [= [(post-BD FEV1 –pre-BD FEV1) / pre-BD FEV1] × 100], *OR (95% CI)* odds ratio and confidence interval. Chi-square for trend, and Kruskal-Wallis tests were used followed by Dunn's multiple comparison test. Bold values indicate statistically significant at *p* < 0.05.

^a^Indicates significant difference from homozygous carriers of the wild allele at *p* < 0.05.

### MIR-196a2 gene polymorphism in relation to bronchodilator response in COPD patients

By applying the linear regression model, genotype was an independent predictor for the bronchodilator response (beta coefficient = 2.306, *p* < 0.001; S5 Table). We found significant differences between the mean values of pre- and post-bronchodilator forced expiratory volume in the first second (FEV1, *p* = 0.012 and *p =* 0.003, respectively) in patients depending on the MIR-196a2 genotypes. In the analysis of mean percentage values of the bronchodilator response in terms of absolute change in FEV1 (BDRABS), change in FEV1 as a percent of predicted FEV1 (BDRPRED) and change in FEV1 as a percent of baseline FEV1 (BDRBASE) to the Salbutamol inhalation in patients stratified by genotypes, we observed under an additive model a significantly (*p* < 0.001) poor responses in COPD patients with CC genotype, an intermediate response in patients with CT genotype and the highest responses in patients with the TT genotype (Table 3). In addition, under an allelic model, T allele carriers showed a favorable response to short-acting β2-agonist in terms of significantly (*p* < 0.001) higher values in previously mentioned pulmonary function tests in comparison to non-carriers (S1 Fig).

## Discussion

**Fig 4:**
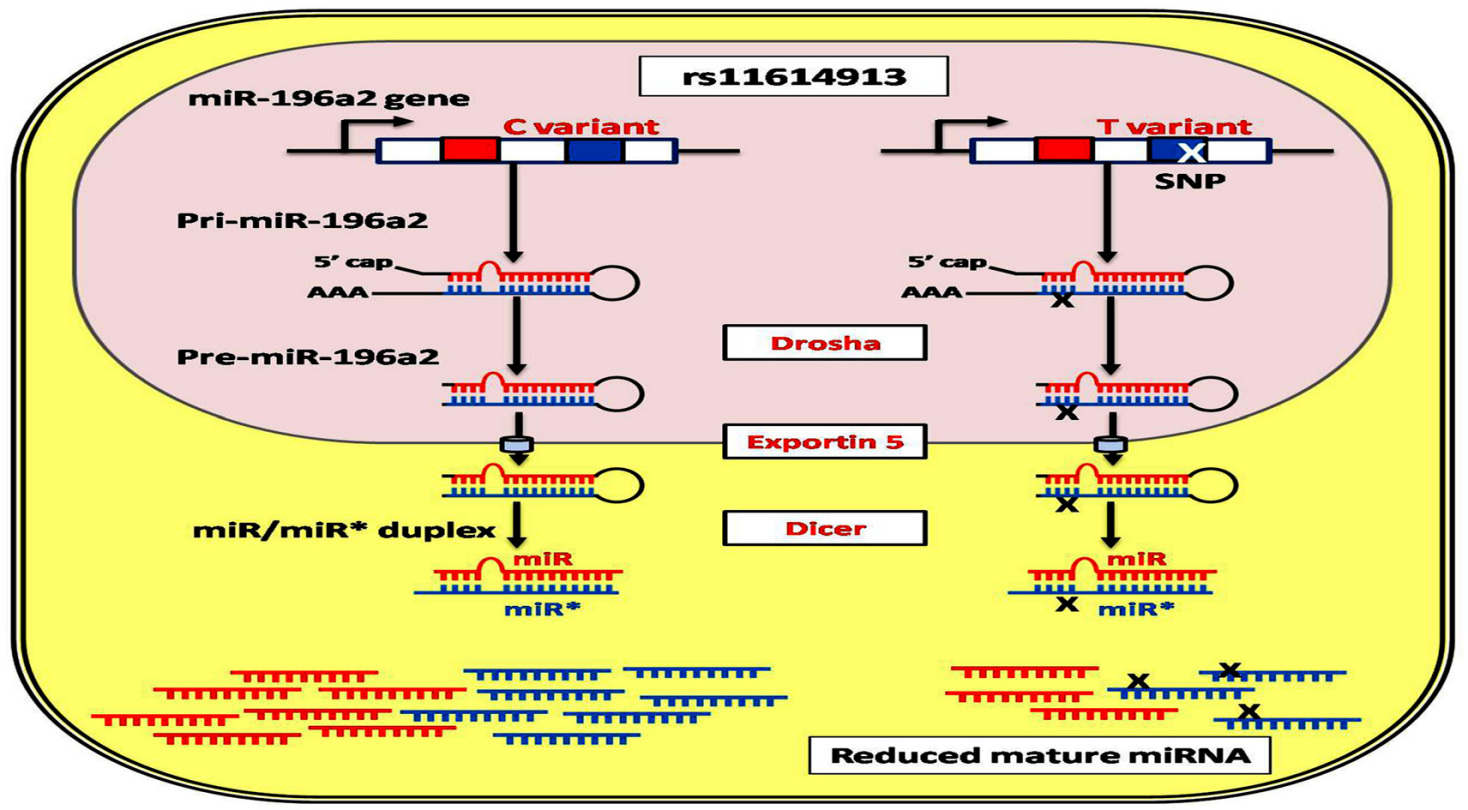
Predicted functional impact of rs11614913 pre-miR-196a2 variant. Hsa-miR-196a-2 is composed of two different mature miRNAs (miR-196a and miR196a*), which are processed from the same stem-loop. The SNP (rs11614913) lies in the mature sequence of miR-196a* but may influence either miRNA by affecting processing of the pre-miRNA to its mature form [47].

MiRNAs are involved in a wide range of biological processes. Small change in the amount of miRNAs may have an effect on thousands of target mRNAs and result in diverse functional consequences. Genetic polymorphisms of miRNAs can potentially influence the processing and maturation of miRNA or their interaction with their mRNA targets. Aberrant expression profile of MIR-196a2 was previously reported in various pulmonary diseases [3]. A common functional genetic variant (rs11614913: C/T) exists in the stem region of the miR-196a2 precursor. Previous studies did not show differential expression levels of both primary and precursor miR-196a2 among different genotypes, however, the higher expression profile of mature miR-196a2 was observed in non-small cell lung tumor samples with CC genotype compared to CT and TT individuals [27]. This evidence was supported by the *in vitro* study of Hoffman *et al*. [47] who reported an increased expression of mature miR-196a2 in breast cancer cells transfected with pre-miR-196a-C vector compared with cells transfected with pre-miR-196a-T, but did not observe the differential expression of the pre-miRNA, suggesting that MIR-196a2 genotypes may result in altered processing of the precursor hairpin loop into mature forms. Interestingly, in our lab we previously found that *rs11614913* (C/T) polymorphism has a significant impact on expression levels of the mature miRNA, with the C allele carriers exhibited higher levels more than two folds in normal and cancer individuals [data not published]. Although there is a lack of similar evidence in specific cell lines and COPD lung tissues, miR-196a2-mediated mechanisms regulating cancer and inflammation were shown to be tightly linked with nearly the same gene regulatory networks implicated in innate and adaptive immune responses [48]. Taken these together with our *in silico* results that predicted the lack of discrepancy in the target gene sets for C and T alleles, we deduce that the studied SNP may cause quantitative rather than qualitative effects on the mature functioning forms of miR-196a2 (Fig 4). Further investigations to study the effect of the rs11614913 SNP on the efficiency of miRNA biogenesis machinery and miRNA-RNA binding protein interactions are mandatory to explore the molecular mechanism underlying the observed differential processing of miR-196a2 [49].

In the present study, network analysis demonstrated that miR-196a2 target a network of several key molecules in COPD pathogenesis which are previously validated by experimental studies. They include *GSTP1* (glutathione S-transferase p1), *ACE* (angiotensin 1 converting enzyme), *HTR4* (hydroxytryptamine receptor4), *PPT2* (palmitoyl-protein thioesterase 2), and *THSD4* (thrombospondin, type I, domain containing 4) genes which are involved in cell-to-cell signaling, immune cell trafficking, and inflammation. Recruitment of inflammatory cells as neutrophils, macrophages and T-lymphocytes, in turn, increases the production of free radicals, oxidative stress and protease function in the lungs of COPD patients [50]. MiR-196a2 also targets *COL3A1* (collagen type 3 alpha 1), *COL1A1*, *COL1A2*, *PDGFRA* (platelet-derived growth factor receptor alpha polypeptide), *EPB41L2* (erythrocyte membrane protein band 4.1-like 2), and *CASK* (calcium/calmodulin-dependent serine protein kinase) genes essential for cell junction and communication. In addition, it is implicated in genes involved in apoptosis via mTOR signaling pathway and proteolysis as *RICTOR* (Rapamycin-insensitive companion of mTOR), *MMP23B* (matrix metalloproteinases 23 B) and *MAPK* (mitogen-activated protein kinase), those two pathways are intensively involved in extracellular matrix degradation and emphysematous parenchymal destruction in COPD. Moreover, *HOXA5*, *HOXA7*, *HOXB6*, *HOXB8*, *and HOXC8* genes, one of the most predicted targets of miR-196a2, are known to regulate lung development. Remarkably, Ning et al. [51] reported down-regulation 5-folds of *COL1A1* and *PDGFRA* in COPD patients with advanced COPD compared to non-obstructive smokers.

Interestingly, the integrin genes, *ITGAV* (integrin alpha V) and *ITGB1* (integrin beta 1) involved in collagen attachment, cytoskeletal remodeling and resolution of damaged tissue, *ROCK1* (Rho-associated coiled-coil containing protein kinase 1) gene that regulate leukocyte trans-endothelial migration and focal adhesion, and several members of the TGFβ pathway, including *SMAD6* (Human mothers against decapentaplegic homolog 6), *TGFBR2* (transforming growth factor beta receptor 2), and *ACVR2B* (activin A receptor type IIB) mediate a variety of cellular processes, including angiogenesis, extracellular matrix remodeling, fibrosis, cell differentiation and immune response, all are targeted by miR-196a2. Campbell et al. [44] reported down-regulation of these genes in COPD and their association with increasing emphysema severity in lung tissue, thus their inhibition by miR-196a2 might be responsible for recruitment of inflammatory cells and cytoskeletal changes in COPD, leading to emphysematous destruction of the lung.

Here in this study, genotype distribution of MIR-196a2 rs11614913 polymorphism was in agreement with that expected under the Hardy-Weinberg equilibrium, but none of the genotypes was associated with increased risk of COPD or disease outcome and severity in our studied patients. However, a Chinese group reported that TT genotype and T allele of MIR-196a2 rs11614913 were significantly associated with a decreased risk for COPD, compared with the CC genotype and the C allele [52]. Other investigators revealed an association between the CC homozygote of rs11614913 SNP and a higher risk to develop lung cancer, one of the most frequent comorbidities and cause of death in patients with COPD [27, 53]. COPD is a complex respiratory system disease with phenotypic heterogeneity (chronic bronchitis and/or emphysema spectrum), therefore highlighting the diversity of potential mechanisms underlying the disease. In addition, the interaction of miRNAs and target genes is also complicated; one miRNA may modulate multiple target genes, whereas one target gene may be regulated by various miRNAs simultaneously [54]. Thus, the impact of the SNP rs11614913 on COPD risk and progression may be masked by other deregulated unidentified causal genes that are implicated in the pathogenesis of COPD.

Interestingly, our results showed that rs11614913 polymorphism was significantly associated with BDR to Salbutamol; patients with CC genotype corresponding with the smallest, CT genotype with the intermediate, and TT genotype with the best response. Our pathway analysis using Diana Tools showed that miR-196a2 target molecules essential for the regulation of both calcium signaling and actin cytoskeleton pathways (*PDGFRA*, *ROCK1*, *OCRL* (oculocerebrorenal syndrome of Lowe) and *DIAPH2* (diaphanous homolog 2 genes), which in turn modulate downstream effectors essential for long-term potential, actin polymerization, actinomyosin assembly contraction, and phosphatidylinositol signaling pathway. This may account for the differential effect of MIR-196a2 genotypes on the bronchodilator response of COPD patients in our study. Consistently, various studies over the past two decades clearly support the notion that genetic polymorphisms alter the BDR to inhaled bronchodilators. Further in-depth analysis may hold the key to the development of novel genetic biomarkers that will enable us to personalize bronchodilator therapy.

Considering the important role of hsa-miR-196a2 in various signaling pathways involved in the pathogenesis of COPD, enhancing our understanding of miRNAs-disease interactions would pave the road towards developing new molecular targets for treatment. Although our results were promising, there were also some limitations need to be considered: [i] because our sample size may be considered small and all participants were males, further efforts at replication in large cohorts with both gender and in different ethnic groups are recommended, [ii] there was no control for environmental factors which could interact with our SNP, with lack of stratification of patients according to COPD spectrum, [iii] it is desirable to examine miRNAs SNPs in patients undertaking various medications because reversibility testing might not predict long-term effect of treatment. To the best of our knowledge, our hypothesis generating pilot study is considered the first one to provide evidence that miRNA SNPs can be used as a candidate biomarker for COPD-targeted treatment. Further study is warranted to explore the interaction of miR-196a2 and their predicted target genes associated with COPD pathogenesis.

In Conclusion, the current results suggested the presence of an association between rs11614913 MIR-196a2 polymorphism and bronchodilator response of COPD patients in the studied male Egyptian population. This could provide novel insight into its potential use as a pharmacogenetic marker for COPD male patients.

## Supporting Information

S1 Table Putative target genes of microRNA-196a2 using different databases. (PDF)

S2 Table Functional annotation of putative microRNA-196a2 target genes in KEGG pathways. (PDF)

S3 Table Genes involved in COPD pathogenesis pathways identified by Inguenity pathway analysis software and in literature. (PDF)

S4 Table Predicted target genes of microRNA-196a2 using DIANA-miRPath v2.0 web-server (http://diana.imis.athena-innovation.gr/DianaTools/index.php?r=mirpath/index) and miRTar Human tool (http://miRTar.mbc.nctu.edu.tw/)" that have a putative role in COPD disease. (PDF)

S5 Table Linear regression analysis: predictors of bronchodilator response in COPD patients. (PDF)

S1 Fig Bronchodilator response, according to *hsa-miR-196a2* alleles in COPD patients. (PDF)
