# Supplementary material for: Association of MicroRNA-196a2 Variant with Response to Short-Acting β2-Agonist in COPD: An Egyptian Pilot Study
Source: PLoS One. 2016 Apr 4;11(4):e0152834. doi: 10.1371/journal.pone.0152834 (PMC4820109; doi:10.1371/journal.pone.0152834)
Supplement: S1 Table — (PDF) [file pone.0152834.s001.pdf]

S1 Table. Putative target genes of microRNA-196a2 using different databases [a]

n=749													n=204			
miRNAmap													miRDB			
ABCC12	BAP1	CDAN1	DLG3	FLNA	HOXA9	LRP4	MYO1E	PHC2	RBM16	SLC9A6	TMEM93	WT1	A2ML1	GCFC2	OTUD6B	TMEM161B
ABCC2	BASP1	CDC73	DLG5	FLNB	HOXB3	LRRC17	MYO1F	PHF2	RBM26	SLCO3A1	TNFRSF21	XPO4	ABCB9	GDNF	P2RY1	TMX1
ABCD2	BAT3	CDH23	DLL1	FLRT3	HOXB6	LRRC39	MYO6	PHF20	RBM35A	SMAD1	TNP1	XPO5	ABI2	GGA3	PACRGL	TOX3
ABCE1	BIRC6	CDYL	DMAP1	FN1	HOXB8	LRRC49	MYPN	PHF3	RCC2	SMAD4	TNPO2	YLPM1	ACER2	GGCX	PANK2	TRERF1
ABCG4	BMI1	CENTD3	DMGDH	FOS	HOXC8	LRRC9	NARG1L	PHF6	RDX	SMARCA4	TOX3	ZC3H10	AQP4	GJB4	PARD6B	TRMT10A
ABHD12	BMX	CHD1	DMXL1	FOXP4	HOXD8	LRSAM1	NAV2	PHF8	RER1	SMARCAD1	TPM2	ZC3H6	ARFGEF1	GPCPD1	PBX1	TRMT1L
ABI3BP	BNC2	CHD5	DNAH2	FPGS	HSD17B10	LSS	NCBP1	PIGT	REV3L	SNAG1	TPT1	ZC3H7A	ARHGAP28	GPRASP1	PBX3	TSPAN12
ACADVL	BRD8	CHD9	DNM3	FREM1	HSP90AA1	LTB4DH	NCDN	PIK3CD	RFX3	SNAP91	TRERF1	ZC3HC1	B3GNT7	GRPEL2	PDCL	TSTD3
ACBD3	BRMS1L	CHDH	DNPEP	FRMD5	HSP90AB1	MACF1	NCOR1	PIP5K1B	RHOG	SNCAIP	TRHDE	ZCCHC6	BACH1	GTDC1	PDGFRA	TTC26
ACCN1	BRPF1	CHIA	DOC2A	FRRS1	HSPA4L	MADD	NDST2	PKD2L1	RIC8B	SNTB2	TRIM16	ZDHHC16	BIRC6	GTF2A1	PHF8	UHRF2
ACE	BRSK2	CHRNA7	DOCK11	FRY	HSPE1	MAEA	NEDD1	PKD2L2	RIMS1	SNX1	TRIM42	ZEB1	BRAP	GUF1	PLD1	VAX1
ACLY	BTBD11	CHST1	DOCK5	GABARAP	HSPH1	MAMDC2	NEDD4L	PKNOX2	RNF10	SNX17	TRIM59	ZER1	C18orf25	HABP4	PLN	VSNL1
ACSS2	BZW1	CHSY1	DOLK	GABARAPL2	HTR4	MAN2A2	NEK1	PLA2R1	RNF111	SNX4	TRIP10	ZFAND5	C9orf72	HADH	POLR3D	WDR37
ACTR1A	C10orf137	CKAP5	DPH1	GABRA2	HUWE1	MAOB	NEO1	PLAG1	RNF12	SORCS3	TRNT1	ZFP36L2	CALD1	HDX	PPAPDC2	YIPF6
ACVR2A	C10orf38	CLGN	DPP3	GABRA5	IBRDC3	MAP3K15	NEUROD1	PLCB2	RNF128	SOX5	TRPC3	ZMYM3	CALM1	HMGA1	PPP1R15B	YOD1
ADAM17	C12orf41	CLINT1	DPY19L4	GALE	IBTK	MAP3K2	NFE2L1	PLCH1	RNF145	SPAG9	TRPC4	ZMYM5	CASK	HMGA2	PRELID2	ZCCHC3
ADAMTS9	C12orf48	CLIP1	DST	GATA6	IDE	MAP3K7IP2	NFKBIA	POLD1	RNF19	SPATA6	TRPM3	ZMYND10	CBFA2T3	HOOK1	PRKG1	ZCCHC9
AGBL3	C14orf103	CLPB	DTNA	GBA2	IDH1	MAPK1	NHLH2	POLG	RPS6KA4	SPNS1	TRPS1	ZMYND11	CCDC47	HOXA5	PRTG	ZDHHC21
AGPAT4	C15orf29	CLSPN	DULLARD	GCN1L1	IGF2BP2	MAPK10	NIT2	POMGNT1	RPS6KB2	SPSB3	TSPAN3	ZNF148	CCNJ	HOXA7	PSMD11	ZMYND11
AKAP6	C15orf44	CNKSR2	DUSP15	GDPD2	IKZF2	MAPK13	NOL6	POP1	RQCD1	SPTBN2	TTBK2	ZNF207	CD24	HOXA9	PTGR1	ZNF268
AKTIP	C17orf84	CNTNAP1	DVL2	GFPT2	ILF3	MAPK8IP3	NONO	POU1F1	RTTN	SPTLC1	TTC1	ZNF281	CDKN1B	HOXB6	PTPRG	ZNF385B
ALDH6A1	C1orf112	COG4	DYNC2LI1	GGPS1	ILK	MAPKAP1	NOTCH1	PPM1A	RUFY1	SSR2	TTLL9	ZNF294	CEP350	HOXB7	RANBP2	ZNF473
ALG9	C1orf128	COG8	DYRK2	GGTL3	IMPDH1	MAPRE2	NPAS4	PPP1R3A	RUTBC1	ST3GAL3	TUBA4B	ZNF609	COL1A2	HOXC8	RASGRP1	ZNF512
AMACR	C1orf32	COL14A1	DYRK3	GIT1	ING1	MARCH6	NPEPPS	PPP2R5A	RXFP2	STAG1	TUBAL3	ZNF644	COL24A1	IGDCC4	RAVER2	ZNF850
ANKRD10	C1orf34	COL24A1	DYSF	GIT2	IPO11	MAST4	NR2C1	PPP2R5E	SALL3	STAG2	TUSC2	ZNF76	COL3A1	IGF2BP3	RBM12B	
ANKRD28	C2	COL2A1	DYX1C1	GLE1L	ITGB3BP	MAT2A	NR4A2	PPP3CB	SAMD8	STAM	TXNRD2	ZNRF1	CREBL2	ING5	RBM8A	
ANXA1	C22orf28	COL4A1	EDIL3	GLT8D2	ITPR2	MBD5	NRG1	PPT2	SAPS2	STAU1	TYRO3	ZNRF2	DCAF15	IQCH	RBMS3	
AP1S2	C3orf59	COL5A2	EFTUD1	GNAS	ITSN1	MBD6	NSL1	PRDM11	SAR1B	STAU2	UBAP2	ZW10	DCDC2	IQCJ-SCHIP1	RCC2	
AP3B1	C4orf31	COL9A1	EGFL9	GOLGA4	JAG1	MBNL2	NTN4	PRDM16	SARDH	STK4	UBE2D3	ZZZ3	DENND6A	KCNQ5	RDX	
AP3D1	C6orf125	COPG	EIF1AY	GOLGA7	KCNC2	MCM4	NUMA1	PRKAG1	SCOC	STRAP	UBL7		DICER1	KDM5A	RET	
AP3M1	C6orf134	COPS3	EIF3S10	GPATCH1	KCNC4	MDN1	NUP160	PRKCB1	SDHA	STRBP	UBTF		DIP2A	KIAA1429	RGL2	
APBB3	C6orf165	COPS6	EIF4G1	GPBP1L1	KCNIP4	ME1	NUP35	PRKG1	SEC13	SUMO1	UCKL1		DIS3	KLHL4	RICTOR	
ARAF	C6orf166	COPS7B	EIF4G3	GPM6B	KCNQ4	MEA1	ODZ1	PRKG2	SEC24A	SYTL2	UHRF2		DNAJC10	LARP4	RIOK3	
ARF1	C7orf11	CPD	ELAVL4	GPR137B	KCNQ5	MELK	OLFML2A	PRKRIR	SEC61A2	TAC1	UNC13B		DNM3	LCOR	RLIM	
ARFGEF1	C7orf42	CPNE8	ELMO1	GPS2	KCTD19	MESDC1	ORC5L	PRLR	SEC61B	TAF1A	UNC45A		DOC2A	LIN28B	RNF10	
ARFIP1	C9orf127	CPSF1	ELMO2	GRIA1	KCTD4	MET	OSBPL11	PRPF31	SEL1L2	TAOK1	USP2		DYNC2LI1	LIX1L	RNF5	
ARNTL	C9orf52	CREB3L1	ENTPD5	GRIN1	KDELR2	MGAT4A	OTUD7A	PRPF4B	SEMA3A	TBC1D10A	USP32		EEA1	LOC102724908	RNMT	
ASB3	CA10	CS	EP300	GTF2A1	KHDRBS1	MKRN1	PA2G4	PRPF8	SEMA6A	TBC1D22B	USP47		EEF2K	LRIG2	ROCK1	
ASH2L	CA7	CSNK2A2	EPB41L2	GTF3C2	KIAA0528	MLL5	PACS2	PRSS7	SENP3	TBL3	UTRN		ELAVL4	LRIG3	RPL35A	
ASTN1	CACNA1A	CSPP1	EPHA7	GTF3C3	KIAA1244	MLLT10	PAFAH1B3	PTPLA	SENP6	TBPL1	VDAC1		ELF4	LRP4	RPS27A	
ATG16L2	CACNA2D2	CSRP2BP	EPS15	GTPBP2	KIAA1411	MMP23B	PAK1	PTPRG	SEPT7	TBX15	VEGFA		ELMOD2	LRRC17	RSPO2	
ATG9A	CACNB3	CTNNA1	ERG	GUCY2C	KIAA1539	MOSPD1	PAPPA	PUM2	SETD2	TBX4	VIM		EPHA3	LRRC4B	SCCPDH	
ATP13A2	CALB1	CTNND2	ESCO1	GUF1	KIAA1797	MOV10	PARP6	PUNC	SFRS10	TCERG1	VPS13A		EPHA7	MAP3K1	SCYL2	
ATP13A4	CALD1	CTRC	EYA1	GYS2	KIAA1967	MPDZ	PAX3	PUS3	SFRS11	TCF7L2	VPS13B		ERI2	MAP4K3	SDCBP	
ATP6V0D1	CALU	CTTNBP2	FAM108C1	HACE1	KIF1C	MRC1	PBX1	QARS	SFRS4	TEX9	VPS26A		ERLIN2	MAPK8	SIRT5	
ATP6V1B1	CAMK2G	CUEDC2	FAM110B	HACL1	KIF27	MRC1L1	PCBP4	RAB21	SGK	TH1L	VPS35		EXOC5	MBNL2	SLC25A5	
ATP8A2	CARD11	CXorf45	FAM13C1	HADHA	KIF5B	MRPL33	PCCB	RAB2A	SGPP1	THBS4	VPS37A		EXOC8	MGAT4A	SLC35F4	
ATPBD1C	CASC3	DBC1	FAM45A	HAS3	KLHL13	MRPL39	PCDH1	RAB5A	SLC12A6	THSD4	VPS52		FAM104A	MTRNR2L4	SLC39A9	
ATRNL1	CASK	DBR1	FAM46A	HBP1	KLHL29	MRVI1	PCDH19	RACGAP1	SLC17A7	THSD7A	VSNL1		FAM13C	MYSM1	SLC9A6	
ATXN2	CAV1	DCUN1D1	FAM49B	HDAC3	KLHL6	MSH4	PCNXL2	RAF1	SLC20A2	TIAF1	WAC		FAM154B	NAP1L1	SLC9B2	
ATXN2L	CBX5	DDX19B	FASTK	HECTD1	KPNA4	MSI1	PDCD10	RALA	SLC25A1	TLE1	WDR3		FAM19A5	NEDD4L	SMC3	
ATXN7L2	CCDC100	DEF6	FAT4	HECTD3	KRT83	MTF1	PDCD11	RALBP1	SLC25A13	TLK1	WDR42A		FAS	NOSTRIN	SNAP91	
AXIN2	CCDC109A	DENND1B	FBN1	HECW1	LARP4	MTF2	PDE11A	RANBP3	SLC30A9	TLN1	WDR8		FBXO47	NR2C2	SNX16	
B4GALT3	CCDC19	DENND4A	FBP2	HERC4	LARP6	MTHFD1	PDE6D	RAP1B	SLC35B1	TM9SF2	WDTC1		FGD6	NR6A1	SNX24	
BAAT	CCDC22	DENND4C	FBXO39	HIP2	LATS1	MYC	PDHA1	RARB	SLC35D3	TMCO2	WHSC1L1		FNIP1	NRAS	SPATA6	
BACH2	CCDC47	DEPDC1B	FBXW9	HK1	LCOR	MYH14	PDXDC1	RARS2	SLC4A7	TMEM117	WISP3		FRMD4B	NTN4	SRRT	
BAI1	CCDC53	DGKH	FES	HMGCS1	LHX2	MYH15	PEX5L	RASA1	SLC4A8	TMEM165	WNK3		GABRA2	ODF2L	SULT2A1	
BAI2	CCNI	DHX29	FGF14	HNRPUL1	LIN28B	MYL6B	PFKFB1	RASGEF1A	SLC5A12	TMEM168	WNT2B		GALC	OPA1	SUOX	
BAI3	CCNJ	DHX57	FHL1	HOOK3	LMO7	MYO16	PGD	RASGEF1B	SLC7A4	TMEM39B	WNT9B		GAN	OPA3	SYT9	
BAIAP2	CCNL1	DICER1	FHL2	HOXA1	LPHN3	MYO1C	PHACTR1	RBBP7	SLC7A7	TMEM87A	WSB1		GATA6	OR7D2	TBPL1	

n=295					n=226				n=108		n=23
Targetscan					miRTarBase				Diana		Diana in KEGG
AAK1	CPM	HDX	NTN4	SLC9A6	ABT1	FLNA	MED13	SAP18	ACTR10 (ENSG00000131966)	LARP4 (ENSG00000161813)	ABCC8
ABCB9	CREBL2	HMGA1	OPCML	SMAD6	ACTB	FOXJ3	MPP2	SAR1B	ARFGEF1 (ENSG00000066777)	LIN28B (ENSG00000187772)	ABL2
ABL1	CTDSP2	HMGA2	OSMR	SMARCC1	AHSA1	FOXO1	MRPL35	SBF1	ARHGAP28 (ENSG00000088756)	LIX1L (ENSG00000152022)	ACSL6
ABL2	CTPS	HOOK1	OTUD6B	SMCR7L	ANXA1	FRS2	MSL3	SBNO1	ATRNL1 (ENSG00000107518)	LONP2 (ENSG00000102910)	ACVR2B
ACER2	DCAF15	HOXA5	OTX1	SMCR8	ANXA7	FXR2	MTRF1L	SCN11A	AUH (ENSG00000148090)	LONRF3 (ENSG00000175556)	BIRC6
ACSL6	DCDC2	HOXA7	PACRGL	SMURF1	APP	GGA3	MYCBP2	SH3GL3	BCAT1 (ENSG00000060982)	LRP1B (ENSG00000168702)	CASK
ACTR10	DDX19A	HOXA9	PAPOLG	SNTB2	ARHGAP28	GID8	NAP1L4	SKI	BIRC6 (ENSG00000115760)	LRP4 (ENSG00000134569)	COL1A2
ADCY9	DDX19B	HOXB6	PARD6B	SNX16	ATG16L1	GLMN	ND4	SLC10A7	C1ORF25 (ENSG00000121486)	MAP3K1 (ENSG00000095015)	COL3A1
AFF2	DIP2A	HOXB7	PARP8	SOCS4	ATG9A	GLTP	ND4L	SLC20A1	CASK (ENSG00000147044)	MBNL2 (ENSG00000139793)	DIAPH2
APBA1	DIRC2	HOXB8	PAX7	SOX11	ATP1A1	GLUL	ND5	SLC25A17	CCDC39 (ENSG00000145075)	MRS2 (ENSG00000124532)	EPB41L2
AQP4	DLGAP2	HOXC8	PBX1	SOX12	ATP6	GMFB	NDFIP1	SLC30A6	CCDC47 (ENSG00000108588)	MSH2 (ENSG00000095002)	FLNB
ARFIP2	DNAJC27	HP1BP3	PBX3	SPRED1	ATP6V1B2	GOT2	NHLRC3	SLC6A8	CCNJ (ENSG00000107443)	NAALADL2 (ENSG00000177694)	GABRA2
ARHGAP20	DOCK3	ICOS	PCDH19	SSR1	BACH1	GRIK4	NKX6-1	SMARCAD1	CDYL (ENSG00000153046)	NBPF1,NBPF11 (ENSG00000203836)	IDE
ARHGAP28	DOK6	IGDCC4	PCYT1B	STEAP2	BCL11A	GSTK1	NOTCH2	SMC3	CLEC2B (ENSG00000110852)	NR2C2 (ENSG00000177463)	MAPK8
ARHGEF15	E2F7	IGF1	PDGFRA	SUDS3	BCORL1	HAND1	NR2F6	SMCR7L	COL14A1 (ENSG00000187955)	NR6A1 (ENSG00000148200)	NME4
ARHGEF38	EBF1	IGF2BP1	PEG10	SYNCRIP	BCS1L	HAUS6	NR4A1	SNRPD1	COL1A2 (ENSG00000164692)	NRAS (ENSG00000213281)	NTN4
ATP8B4	EEA1	IGF2BP3	PHF20	SYT9	BIN3	HIST1H2BB	NRBP1	SPATA2	COL3A1 (ENSG00000168542)	OPCML (ENSG00000183715)	PCYT1B
AZI2	EEF2K	IGFBP3	PHOSPHO2-KLHL23	TAOK1	BMP4	HIST2H4B	NRDE2	SPEN	CREBL2 (ENSG00000111269)	OTUD6B (ENSG00000155100)	PDGFRA
B3GNT7	EIF2S2	IMPAD1	PLDN	TEAD3	BRMS1L	HMGA1	NRXN1	SPRR2C	DAZ1 (ENSG00000188120)	PABPC1L2A (ENSG00000186288)	PLOD1
BACH1	ELF4	ING5	PLEKHA8	TGFBR3	BUB1	HMGA2	NUP50	SPRYD4	DAZ2 (ENSG00000205944)	PACRGL (ENSG00000163138)	RBPJ
BCAT1	ELK4	IQCJ-SCHIP1	POLR3D	TMEM143	C11orf57	HMOX1	OAT	SRP9	DAZ3 (ENSG00000187191)	PHF20 (ENSG00000025293)	ROCK1
BCL11A	EPC2	KCNE3	PPAPDC2	TMEM194A	C12orf4	HOXA5	OGFRL1	SRRT	DAZ4 (ENSG00000205916)	PLN (ENSG00000198523)	XYLT2
BEND4	EPHA3	KCNJ2	PPP1R15B	TMOD2	C19orf55	HOXA7	PALLD	STK40	DCDC2 (ENSG00000146038)	POLR3D (ENSG00000168495)	
BIRC6	EPHA7	KCNQ5	PPP1R16B	TMX1	C9orf41	HOXB7	PATL1	SYT9	DDX19B (ENSG00000157349)	PRELID2 (ENSG00000186314)	
BNC2	EPS15	KCTD21	PPP6R2	TOX3	CANX	HOXB8	PCGF3	SYVN1	ENSG00000132017	PTPRG (ENSG00000144724)	
C11orf57	ERG	KDM5A	PRTG	TRANK1	CASP3	HOXC8	PDCD4	TAB2	ENSG00000143674	RASGRP1 (ENSG00000172575)	
C11orf84	ERLIN2	KIAA1274	PRUNE2	TRERF1	CCDC47	HOXD8	PDE6D	TAF15	ENSG00000189238	RASSF3 (ENSG00000153179)	
C14orf147	EXOC5	KLHDC8B	PTPRG	TSC1	CCND1	HUWE1	PEX13	TIMM23	ERG (ENSG00000157554)	RAVER2 (ENSG00000162437)	
C15orf29	EXOC8	KLHL23	RAD23B	TSPAN12	CCND2	IFNGR1	PGAM1	TMEM135	ERI2 (ENSG00000196678)	RBM12B (ENSG00000183808)	
C1GALT1	EYA4	LARP4	RALBP1	TSPAN18	CCNE2	IGDCC4	PHC3	TMEM161B	EXOC5 (ENSG00000070367)	RCC2 (ENSG00000179051)	
C20orf112	FAM102B	LCOR	RANBP10	TTL	CCNT2	IGF1R	PNP	TMEM194A	EXOC8 (ENSG00000116903)	RG9MTD2 (ENSG00000145331)	
C20orf160	FAM104A	LCORL	RANBP2	UBE2G2	CDKN1B	IGF2BP1	POLR2D	TMEM2	FRMD5 (ENSG00000171877)	RGS6 (ENSG00000182732)	
C4orf32	FAM110B	LGALS8	RAP1GAP2	UHRF2	CEP120	IGF2BP3	POTEG	TMX2	GABRA2 (ENSG00000151834)	RNF10 (ENSG00000022840)	
C6orf168	FAM127A	LIN28A	RAPGEF5	USP15	CKAP2L	IKBKB	PROSER1	TP53RK	GALC (ENSG00000054983)	RSPO2 (ENSG00000147655)	
C9orf5	FAM127B	LIN28B	RASGRP1	USP31	CNOT11	IPO5	PRPF8	TRA2B	GAN (ENSG00000127688)	SLC35D2 (ENSG00000130958)	
CALM1	FAM127C	LOR	RASSF3	VSNL1	COPS3	ITGAV	PRUNE2	TRAP1	GATA6 (ENSG00000141448)	SLC9A2 (ENSG00000115616)	
CALM3	FAM169A	LRIG2	RAVER2	WDR37	COX3	KATNAL1	PSMC3	TRAPPC9	GDF3 (ENSG00000184344)	SLC9A6 (ENSG00000198689)	
CASK	FAM178A	LRIG3	RCC2	WIPF1	CPD	KCTD1	PSMD8	TRPC3	HAND1 (ENSG00000113196)	SNX13 (ENSG00000071189)	
CBFA2T3	FAM19A5	LRP1B	RGL2	YIPF6	CPEB3	KIAA1804	RAB21	TSKU	HDAC9 (ENSG00000048052)	SNX24 (ENSG00000064652)	
CCDC47	FAM55C	LRRC4B	RGS6	YOD1	DFFA	KIF18B	RAB31	TSPAN12	HMGA1 (ENSG00000137309)	SORCS1 (ENSG00000108018)	
CCNJ	FBXO45	LRRTM3	RICTOR	ZBTB39	DIEXF	KLHL7	RAB7L1	TUBA1B	HMGA2 (ENSG00000149948)	SSR1 (ENSG00000124783)	
CDC25A	FGF14	LTN1	RIOK3	ZCCHC3	DNTTIP2	KMT2C	RAD9A	TUBB	HOXA5 (ENSG00000106004)	SULT2A1 (ENSG00000105398)	
CDC34	FLRT1	MAGT1	RNF5	ZDHHC21	DYRK2	KPNA5	RALGPS2	U2AF2	HOXA7 (ENSG00000122592)	TOX3 (ENSG00000103460)	
CDC73	FNIP1	MAP3K1	RPGRIP1L	ZMYND11	ECHDC1	KRT5	RANBP9	UBE2C	HOXA9 (ENSG00000078399)	TRERF1 (ENSG00000124496)	
CDKN1B	FOXO1	MAP4K3	RSPO2	ZNF148	EEF2	LBR	RASSF7	UBE2Z	HOXB6 (ENSG00000108511)	TRPC3 (ENSG00000138741)	
CDV3	FOXP2	MARS2	RXFP2	ZNF200	EIF2B4	LGR4	RBMX	UQCRC2	HOXB7 (ENSG00000120087)	TXNDC6 (ENSG00000181322)	
CDYL	GALC	MBNL2	SAP30L	ZNF248	EIF2S3	LIN28B	RDH10	USP19	HOXC8 (ENSG00000037965)	UGT2A1 (ENSG00000173610)	
CECR6	GAN	MGAT4A	SCHIP1	ZNF354A	ENAH	LLGL1	RDH11	USP24	IGF2BP1 (ENSG00000159217)	VSNL1 (ENSG00000163032)	
CELF2	GAS7	MRS2	SCRT1	ZNF507	EPHA7	LRP2	REEP2	VCL	IGF2BP3 (ENSG00000136231)	WDR78 (ENSG00000152763)	
CEP350	GATA6	MTMR3	SCRT2	ZNF512	ESPL1	LRRC41	RFC2	VCP	ING5 (ENSG00000168395)	XAF1 (ENSG00000132530)	
CHRD	GCNT4	NAP1L1	SEMA3A	ZNF516	ETV3	LSM14A	RFX5	VDAC2	KCNJ2 (ENSG00000123700)	ZDHHC21 (ENSG00000175893)	
CLCN5	GLG1	NCS1	SERP1	ZNF652	EWSR1	LSM3	RPS2	VDAC3	KCNQ5 (ENSG00000185760)	ZMYND11 (ENSG00000015171)	
CNTRL	GLTP	NEDD4L	SETD8	ZNF689	FAM104A	LYRM2	RPUSD2	ZBTB24	KLHL23 (ENSG00000213160)	ZNF24 (ENSG00000172466)	
COL14A1	GPCPD1	NEURL1B	SFMBT1	ZNF710	FAM127A	MAP4	RUFY2	ZBTB6	KLHL4 (ENSG00000102271)		
COL1A1	GPR156	NHS	SLC25A22	ZNF81	FKTN	MED12	S100A9	ZCCHC11	KLRB1 (ENSG00000111796)		
COL1A2	GRPEL2	NME4	SLC2A12					ZFP64			
COL24A1	GSPT1	NR2C2	SLC30A6					ZNF354B			
COL3A1	GYG2	NRAS	SLC31A1					ZNF398			
CORIN	HABP4	NRK	SLC35D2					ZNF529			
CPD	HAND1	NRXN1	SLC8A2					ZNF581			

[a] Yellow shaded cells are genes involved in COPD pathogenesis [14, 40 – 42].
