# Supplementary material for: Association of MicroRNA-196a2 Variant with Response to Short-Acting β2-Agonist in COPD: An Egyptian Pilot Study
Source: PLoS One. 2016 Apr 4;11(4):e0152834. doi: 10.1371/journal.pone.0152834 (PMC4820109; doi:10.1371/journal.pone.0152834)
Supplement: S2 Table — (PDF) [file pone.0152834.s002.pdf]

S2 Table. Functional annotation of putative microRNA-196a2 target genes in KEGG pathways

KEGG Pathway	Gene Name	Found Genes	-ln(p-value)	Ensembl Gene ID	KEGG Pathway ID
Focal adhesion	MAPK8, PDGFRA, FLNB, ROCK1, COL3A1, COL1A2	6	12.14	ENSG00000107643, ENSG00000134853, ENSG00000136068, ENSG00000067900, ENSG00000168542, ENSG00000164692	hsa04510
Type II diabetes mellitus	MAPK8, ABCC8	2	5.24	ENSG00000107643, ENSG00000006071	hsa04930
Adipocytokine signaling pathway	MAPK8, ACSL6	2	2.7	ENSG00000107643, ENSG00000164398	hsa04920
ECM-receptor interaction	COL3A1, COL1A2	2	2.27	ENSG00000168542, ENSG00000164692	hsa04512
Colorectal cancer	MAPK8, PDGFRA	2	2.09	ENSG00000107643, ENSG00000134853	hsa05210
ErbB signaling pathway	MAPK8, ABL2	2	2.02	ENSG00000107643, ENSG00000143322	hsa04012
TGF-beta signaling pathway	ROCK1, ACVR2B	2	1.96	ENSG00000067900, ENSG00000114739	hsa04350
Regulation of actin cytoskeleton	PDGFRA, ROCK1, DIAPH2	3	1.77	ENSG00000134853, ENSG00000067900, ENSG00000147202	hsa04810
Aminophosphonate metabolism	PCYT1B	1	1.69	ENSG00000102230	hsa00440
Chondroitin sulfate biosynthesis	XYLT2	1	1.29	ENSG00000015532	hsa00532
MAPK signaling pathway	MAPK8, PDGFRA, FLNB	3	1.22	ENSG00000107643, ENSG00000134853, ENSG00000136068	hsa04010
Axon guidance	NTN4, ROCK1	2	1.22	ENSG00000074527, ENSG00000067900	hsa04360
Tight junction	EPB41L2, CASK	2	1.06	ENSG00000079819, ENSG00000147044	hsa04530
Cell Communication	COL3A1, COL1A2	2	1.03	ENSG00000168542, ENSG00000164692	hsa01430
Alzheimer's disease	IDE	1	1.02	ENSG00000119912	hsa05010
Wnt signaling pathway	MAPK8, ROCK1	2	0.9	ENSG00000107643, ENSG00000067900	hsa04310
Lysine degradation	PLOD1	1	0.64	ENSG00000083444	hsa00310
Notch signaling pathway	RBPJ	1	0.59	ENSG00000168214	hsa04330
ABC transporters - General	ABCC8	1	0.55	ENSG00000006071	hsa02010
Fatty acid metabolism	ACSL6	1	0.49	ENSG00000164398	hsa00071
Pathogenic Escherichia coli infection - EHEC	ROCK1	1	0.4	ENSG00000067900	hsa05130
Pathogenic Escherichia coli infection - EPEC	ROCK1	1	0.4	ENSG00000067900	hsa05131
Calcium signaling pathway	PDGFRA	1	0.38	ENSG00000134853	hsa04020
Purine metabolism	NME4	1	0.26	ENSG00000103200	hsa00230
Insulin signaling pathway	MAPK8	1	0.25	ENSG00000107643	hsa04910
Glioma	PDGFRA	1	0.23	ENSG00000134853	hsa05214
Epithelial cell signaling in Helicobacter pylori	MAPK8	1	0.21	ENSG00000107643	hsa05120
Ubiquitin mediated proteolysis	BIRC6	1	0.2	ENSG00000115760	hsa04120
Glycerophospholipid metabolism	PCYT1B	1	0.19	ENSG00000102230	hsa00564
PPAR signaling pathway	ACSL6	1	0.17	ENSG00000164398	hsa03320
Glycan structures - biosynthesis 1	XYLT2	1	0.15	ENSG00000015532	hsa01030
Melanoma	PDGFRA	1	0.14	ENSG00000134853	hsa05218
Pancreatic cancer	MAPK8	1	0.14	ENSG00000107643	hsa05212
Fc epsilon RI signaling pathway	MAPK8	1	0.12	ENSG00000107643	hsa04664
Neuroactive ligand-receptor interaction	GABRA2	1	0.11	ENSG00000151834	hsa04080
Cytokine-cytokine receptor interaction	PDGFRA, ACVR2B	2	0.11	ENSG00000134853, ENSG00000114739	hsa04060
Leukocyte transendothelial migration	ROCK1	1	0.1	ENSG00000067900	hsa04670
Pyrimidine metabolism	NME4	1	0.05	ENSG00000103200	hsa00240
Toll-like receptor signaling pathway	MAPK8	1	0.05	ENSG00000107643	hsa04620
Prostate cancer	PDGFRA	1	0.02	ENSG00000134853	hsa05215
GnRH signaling pathway	MAPK8	1	0.01	ENSG00000107643	hsa04912
Gap junction	PDGFRA	1	0	ENSG00000134853	hsa04540
