# Supplementary material for: Association of MicroRNA-196a2 Variant with Response to Short-Acting β2-Agonist in COPD: An Egyptian Pilot Study
Source: PLoS One. 2016 Apr 4;11(4):e0152834. doi: 10.1371/journal.pone.0152834 (PMC4820109; doi:10.1371/journal.pone.0152834)
Supplement: S3 Table — (PDF) [file pone.0152834.s003.pdf]

S3 Table. Genes involved in COPD pathogenesis pathways identified by Inguenity pathway analysis software and in literature [a]

n=58		n=18	n=21	n=25
COPD genes by Kaneko 2013 (IPA software)		COPD genes by Campbell 2012	COPD genes by Ezzei 2012	COPD genes by Chen 2008
ABCC1	INTS12	ACVRL1	ACVR1	AKR1B1
ACE	IREB2	BCL11A	AGBL1	AKR1B10
ADAM19	LEP	BMPR2	BMP7	CDC2L1
ADAM33	MACROD2	ENG	E2F4	CDKN1A
ADRB2	MMP1	EPAS1 (HIF-2a)	GDF10	cICAM-1
AGER	MMP12	GATA2	INHBB	COL1A1
BICD1	MMP9	ITGB1	LTBP1	CP
CHRNA3	MSR1	KLF13	MAF	CTGF
CHRNA5	NFkBIB	SMAD1	MYH11	CX3CL1
CNTN5	NOS3	SMAD2	PLN	CXCL8
CSF2	NPNT	SMAD3	PPP1R3C	CXCR1
CTLA4	PDE4D	SMAD6	ROCK1	CXCR2
CYP2A6	PPT2	SMAD7	SAPS1	CXCR3
DBP	SERPINA1	TAL1	SFTPA1B	CXCR8
EDN1	SERPINE2	TBX3	SLIT2	CYP1B1
EPHX1	SFTPB	TGFB	SMAD7	CYR61
ESR1	SFTPD	TGFBR1	SMURF1	ERG
FAM13A	SIRT2	TGFBR2	SOSTDC1	IGFBP5
FGF7	SOD3		TGFBR1	LUM
GC	STAT1		TGFBR2	MGC11242
GSTCD	TGFB1		THBS1	MMP7
GSTM1	THSD4			PDGFRA
GSTO2	TLR4			SOD2
GSTP1	TNFA			TGFB1
HHIP	TNS1			TGFBR3
HMOX1	TP53			
HTR4	TRPV4			
IL13	XRCC1			
IL1B				
IL6				

[a] Yellow shaded cells are miR-196a2 target genes.
