# Supplementary material for: Association of MicroRNA-196a2 Variant with Response to Short-Acting β2-Agonist in COPD: An Egyptian Pilot Study
Source: PLoS One. 2016 Apr 4;11(4):e0152834. doi: 10.1371/journal.pone.0152834 (PMC4820109; doi:10.1371/journal.pone.0152834)
Supplement: S4 Table — (PDF) [file pone.0152834.s004.pdf]

S4 Table. Predicted target genes of microRNA-196a2 using DIANA-miRPath v2.0 web-server (http://diana.imis.athena-innovation.gr/DianaTools/index.php?r=mirpath/index) and miRTar Human tool (http://miRTar .mbc.nctu.edu.tw/)" that have a putative role in COPD disease

Gene ID	Gene title	Gene symbol	Function and signaling pathway
ENSG00000159640	angiotensin 1 converting enzyme	ACE	Cell-to-cell signaling and interaction, hematological system development and function, immune cell trafficking
ENSG00000114739	activin A receptor type IIB	ACVR2B	Cytokine-cytokine receptor interaction, TGF-beta signaling
ENSG00000000861	B-cell CLL/lymphoma 11A	BCL11A	Transcription regulation
ENSG00000147044	calcium/calmodulin-dependent serine protein	CASK	Cell junction and communication
ENSG00000108821	collagen type 1 alpha 1	COL1A1	ECM-receptor interaction, connectiuve tissues and cartilage formation
ENSG00000164692	collagen type 1 alpha 2	COL1A2	Cell junction and communication, ECM-receptor interaction
ENSG00000168542	collagen type 3 alpha 1	COL3A1	Cell junction and communication, focal adhesion, ECM-receptor interaction
ENSG00000147202	diaphanous homolog 2	DIAPH2	Regulation of actin cytoskeleton
ENSG00000079819	erythrocyte membrane protein band 4.1-like 2	EPB41L2	Cell junction and communication, regulation of actin cytoskeleton
ENSG00000157554	erythroblast transformation- specific	ERG	Transcription regulation
ENSG00000136068	Filamin B, beta (actin binding protein 278)	FLNB	MAPK signaling pathway, focal adhesions, regulation of actin cytoskeleton
ENSG00000084207	glutathione S-transferase pi 1	GSTP1	Aryl hydrocarbon receptor signaling: Inflammatory disease, respiratory disease, cellular movement
ENSG00000106004	homeobox A5	HOXA5	Lung development, transcription factor
ENSG00000122592	homeobox A7	HOXA7	Lung development
ENSG00000108511	homeobox B6	HOXB6	Lung development
ENSG00000120068	homeobox B8	HOX8B	Lung development
ENSG00000037965	homeobox C8	HOXC8	Lung development
ENSG00000164270	5-hydroxytryptamine (serotonin) receptor 4	HTR4	Neurological disease, nutritional disease, psychological disorders
ENSG00000138448	integrin alpha V	ITGAV	Regulation of actin cytoskeleton, ECM-receptor interaction, signal transduction
ENSG00000150093	integrin beta 1	ITGB1	Regulation of actin cytoskeleton, ECM-receptor interaction,hemostasis, tissue repair, immune response
ENSG00000107643	mitogen-activated protein kinase	MAPK8	Focal adhesion, epithelial cell signaling, wnt signaling, Toll-like receptor signaling, apoptosis
ENSG00000189409	matrix metalloproteinases 23 B	MMP23B	mTOR signaling pathway, tissue remodeling
ENSG00000122126	oculocerebrorenal syndrome of Lowe	OCRL	Regulation of actin cytoskeleton
ENSG00000134853	platelet-derived growth factor receptor alpha polypeptide	PDGFRA	Cell junction and communication, cytokine-cytokine receptor interaction, calcium signaling, MAPK signaling
ENSG00000198523	phospholamban	PLN	Muscle contraction and action potential
ENSG00000168452	palmitoyl-protein thioesterase 2	PPT2	Cell death and survival, cell cycle, cellular movement
ENSG00000164327	Rapamycin-insensitive companion of mammalian target of rapamycin	RICTOR	mTOR signaling pathway, cell growth
ENSG00000067900	Rho-associated, coiled-coil containing protein kinase 1	ROCK1	Leukocyte trans-endothelial migration, regulation of actin cytoskeleton, Wnt signaling, TGF-beta signaling
ENSG00000170365	Human mothers against decapentaplegic homolog 1	SMAD1	TGF-beta signaling, cell growth, apoptosis, morphogenesis, development and immune responses
ENSG00000137834	Human mothers against decapentaplegic homolog 6	SMAD6	TGF-beta/activin-signalling, BMP signaling
ENSG00000163513	transforming growth factor beta receptor 2	TGFBR2	TGF-beta signaling, cell proliferation, differentiation and death
ENSG00000069702	transforming growth factor beta receptor 3	TGFBR3	TGF-beta signaling, tissue fibrosis
ENSG00000187720	thrombospondin, type I, domain containing 4	THSD4	Cell death and survival, cell cycle, cellular movement, microfibril assembly
