# Supplementary material for: Association of MicroRNA-196a2 Variant with Response to Short-Acting β2-Agonist in COPD: An Egyptian Pilot Study
Source: PLoS One. 2016 Apr 4;11(4):e0152834. doi: 10.1371/journal.pone.0152834 (PMC4820109; doi:10.1371/journal.pone.0152834)
Supplement: S5 Table — (PDF) [file pone.0152834.s005.pdf]

S5 Table. Linear regression analysis: predictors of bronchodilator response in COPD patients.

Factors entered into the model were rs11614913 genotypes, age, body mass index, smoking status, family history of COPD, CAT score, mMRC scale, and GOLD stage. Genotype, age, smoking, and family history were independent predictors of bronchodilator response (BDRBASE). This response is defined as a change in FEV1 as a percent of baseline FEV1 [= ((post-BD FEV1 – pre-BD FEV1) / pre-BD FEV1) × 100].

**Independent variables**	**β**	**SE**	***p***	**95% CI**
**(Constant)**	-5.289	2.939	0.075	(-11.1-0.541)
**Genotype**	2.306	0.514	<0.001	(1.28-3.32)
**Smoking**	0.919	0.539	0.091	(-0.15-1.98)
**Age**	0.138	0.051	0.008	(0.03-0.23)
**Family history**	-1.269	0.710	0.077	(-2.67-0.13)

β, Regression coefficients; SE, Standard error; *p*, significant value < 0.05; CI, confidence interval
