# Supplementary material for: Association of MicroRNA-196a2 Variant with Response to Short-Acting β2-Agonist in COPD: An Egyptian Pilot Study
Source: PLoS One. 2016 Apr 4;11(4):e0152834. doi: 10.1371/journal.pone.0152834 (PMC4820109; doi:10.1371/journal.pone.0152834)
Supplement: S1 Fig — (PDF) [file pone.0152834.s006.pdf]

(A) (B)

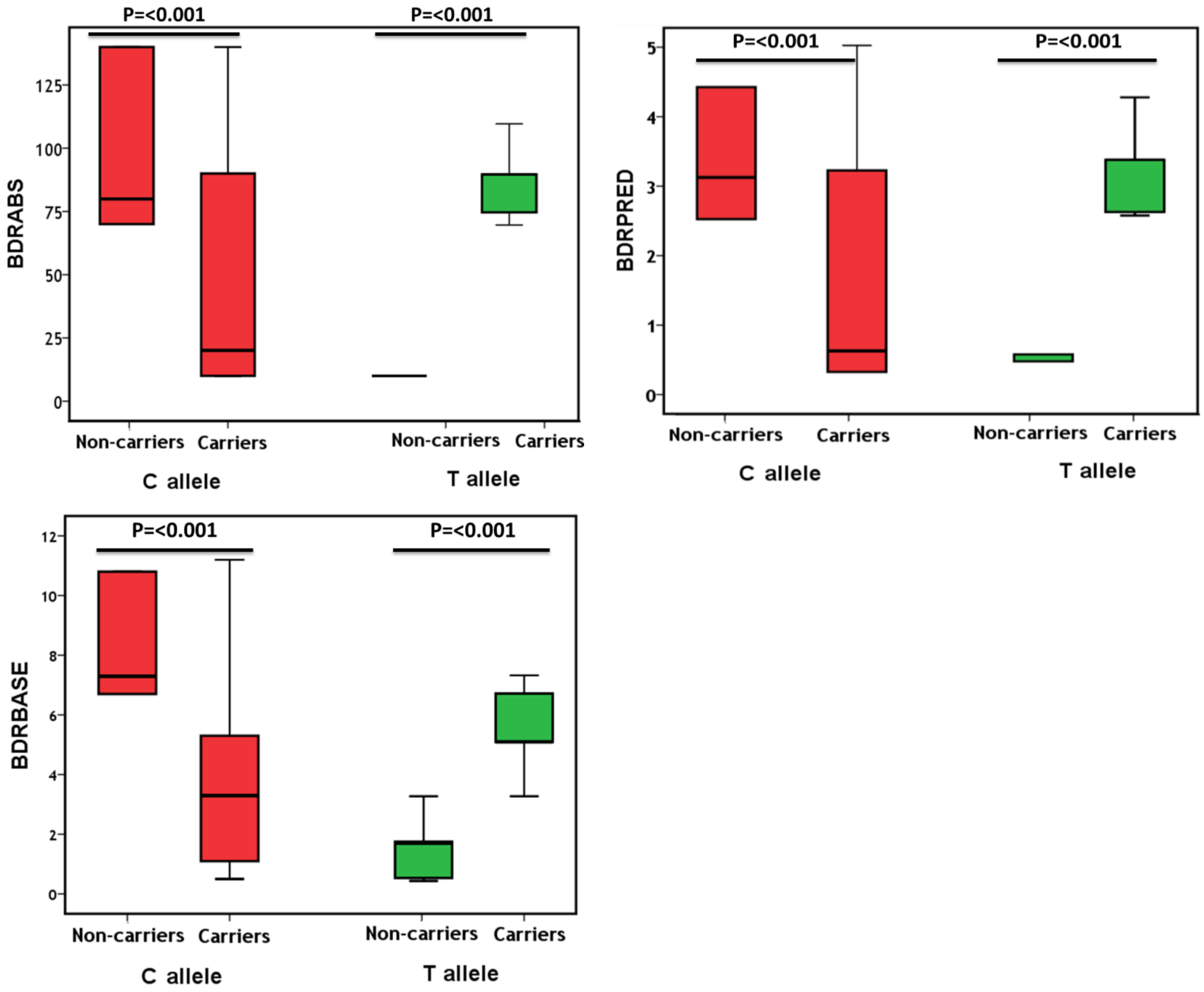

(C)

S1 Fig. Bronchodilator response, according to *hsa-miR-196a*2 alleles in COPD patients. Data are represented as medians. The box defines upper and lower quartiles (25% and 75%, respectively) and the error bars indicate upper and lower adjacent limits. Mann-Whitney test was used. BDRABS absolute change in forced expiratory volume in the first second (FEV1), BDRPRED change in FEV1 as a percent of predicted FEV1, BDRBASE change in FEV1 as a percent of baseline FEV1, all calculations have been explained in detail in the text.

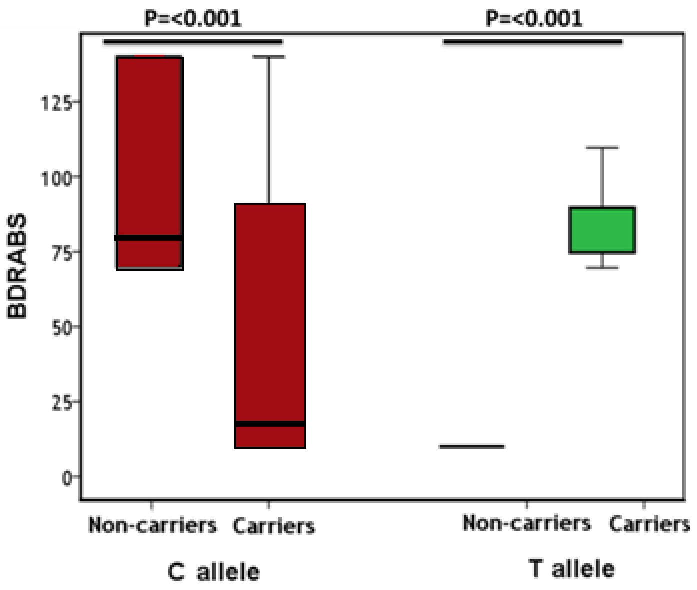
P=<0.001
P=<0.001
BDRABS
0
25
50
75
100
125
Non-carriers
Carriers
Non-carriers
Carriers
C allele
T allele

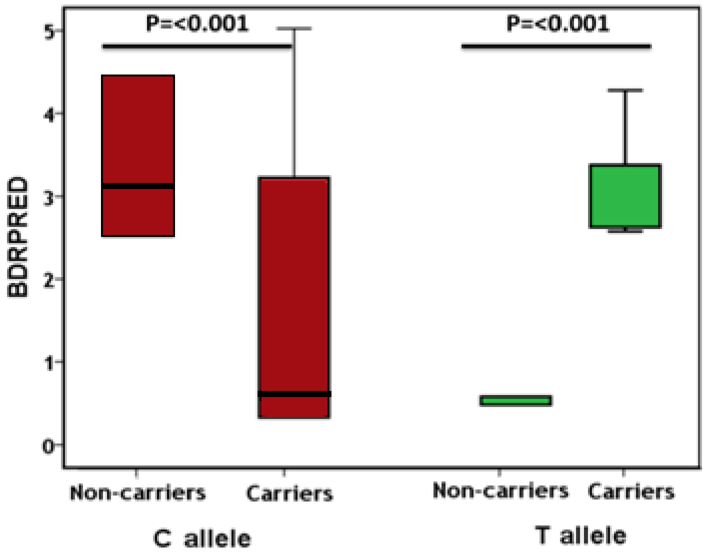
P=<0.001
P=<0.001
BDRPRED
0
1
2
3
4
5
Non-carriers
Carriers
Non-carriers
Carriers
C allele
T allele

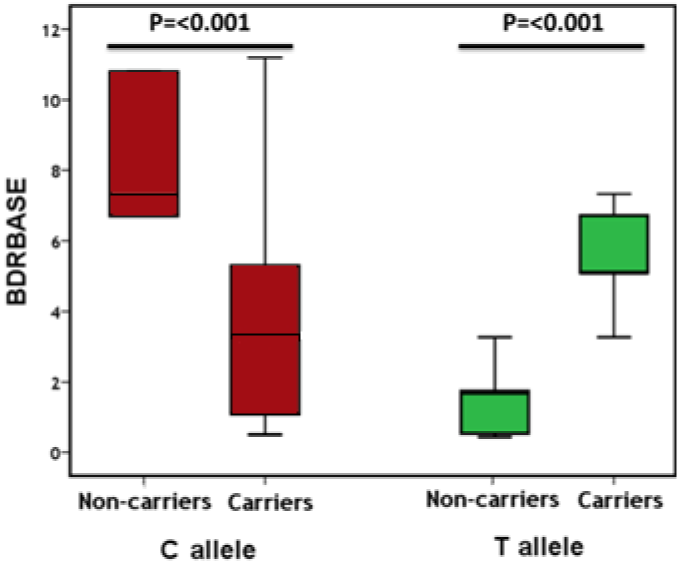
P=<0.001
P=<0.001
BDRBASE
12
10
8
6
4
2
0
Non-carriers
Carriers
C allele
Non-carriers
Carriers
T allele
